# Self-Enhancement of Hepatitis C Virus Replication by Promotion of Specific Sphingolipid Biosynthesis

**DOI:** 10.1371/journal.ppat.1002860

**Published:** 2012-08-16

**Authors:** Yuichi Hirata, Kazutaka Ikeda, Masayuki Sudoh, Yuko Tokunaga, Akemi Suzuki, Leiyun Weng, Masatoshi Ohta, Yoshimi Tobita, Ken Okano, Kazuhisa Ozeki, Kenichi Kawasaki, Takuo Tsukuda, Asao Katsume, Yuko Aoki, Takuya Umehara, Satoshi Sekiguchi, Tetsuya Toyoda, Kunitada Shimotohno, Tomoyoshi Soga, Masahiro Nishijima, Ryo Taguchi, Michinori Kohara

**Affiliations:** 1 Department of Microbiology and Cell Biology, Tokyo Metropolitan Institute of Medical Science, Setagaya-ku, Tokyo, Japan; 2 Department of Metabolome, Graduate School of Medicine, The University of Tokyo, Bunkyo-ku, Tokyo, Japan; 3 Institute for Advanced Biosciences, Keio University, Kakuganji, Tsuruoka, Yamagata, Japan; 4 Kamakura Research Laboratories, Chugai Pharmaceutical Co., Ltd., Kamakura, Kanagawa, Japan; 5 Institute of Glycoscience, Tokai University, Hiratsuka-shi, Kanagawa, Japan; 6 Unit of Viral Genome Regulation, Institut Pasteur of Shanghai, Key Laboratory of Molecular Virology & Immunology, Chinese Academy of Sciences, Shanghai, China; 7 Fuji-Gotemba Research Laboratories, Chugai Pharmaceutical Co., Ltd., Gotemba, Shizuoka, Japan; 8 Research Institute, Chiba Institute of Technology, Narashino, Chiba, Japan; 9 National Institute of Health Sciences, Setagaya-ku, Tokyo, Japan; 10 Showa Pharmaceutical University, Machidashi, Tokyo, Japan; 11 Department of Biomedical Sciences, College of Life and Health Sciences, Chubu University, Kasugai-shi, Aichi, Japan; University of California, San Diego, United States of America

## Abstract

Lipids are key components in the viral life cycle that affect host-pathogen interactions. In this study, we investigated the effect of HCV infection on sphingolipid metabolism, especially on endogenous SM levels, and the relationship between HCV replication and endogenous SM molecular species. We demonstrated that HCV induces the expression of the genes (*SGMS1* and *2*) encoding human SM synthases 1 and 2. We observed associated increases of both total and individual sphingolipid molecular species, as assessed in human hepatocytes and in the detergent-resistant membrane (DRM) fraction in which HCV replicates. SGMS1 expression had a correlation with HCV replication. Inhibition of sphingolipid biosynthesis with a hepatotropic serine palmitoyltransferase (SPT) inhibitor, NA808, suppressed HCV-RNA production while also interfering with sphingolipid metabolism. Further, we identified the SM molecular species that comprise the DRM fraction and demonstrated that these endogenous SM species interacted with HCV nonstructural 5B polymerase to enhance viral replication. Our results reveal that HCV alters sphingolipid metabolism to promote viral replication, providing new insights into the formation of the HCV replication complex and the involvement of host lipids in the HCV life cycle.

## Author Summary

One of the key components for hepatitis C virus (HCV) propagation is lipids, some of which comprise membranous replication complexes for HCV replication. Research on cofactors that are involved in the formation of the membranous replication complex has advanced steadily; on the other hand, the lipids constituting the membranous replication complex remain to be elucidated. Here, we report that HCV modulates sphingolipid metabolism by promoting sphingolipid biosynthesis, to enhance viral replication. Specifically a specific molecular species of sphingomyelin (SM), a type of sphingolipid interacts with HCV nonstructural 5B polymerase, enhancing HCV replication. This work highlights the relationship between specific molecular species of SMs and HCV replication, giving new insight into the formation of the HCV replication complex and the involvement of host lipids in the HCV life cycle.

## Introduction

Lipids have long been known to play dual roles in biological systems, functioning in structural (in biological membranes) and energy storage (in cellular lipid droplets and plasma lipoproteins) capacities. Research over the past few decades has identified additional functions of lipids related to cellular signaling, microdomain organization, and membrane traffic. There are also strong indications of the important role of lipids in various stages of host-pathogen interactions [1].

Sphingomyelin (SM) is a sphingolipid that interacts with cholesterol and glycosphingolipid during formation of the raft domain, which can be extracted for study as a detergent-resistant membrane (DRM) fraction [2]. Recently, raft domains have drawn attention as potential platforms for signal transduction and pathogen infection processes [3], [4]. For instance, raft domains may serve as sites for hepatitis C virus (HCV) replication [5], [6]. Additionally, *in vitro* analysis indicates that synthetic SM binds to the nonstructural 5B polymerase (RdRp) of HCV [7]. This association allows RdRp to localize to the DRM fraction (known to be the site of HCV replication) and activates RdRp, although the degree of binding and activation differs among HCV genotypes [7], [8]. Indeed, suppression of SM biosynthesis with a serine palmitoyltransferase (SPT) inhibitor disrupts the association between RdRp and SM in the DRM fraction, resulting in the suppression of HCV replication [7], [9].

Multiple reports have indicated that HCV modulates lipid metabolism (e.g., cholesterol and fatty acid biosynthesis) to promote viral replication [10]–[12]. However, the effect of HCV infection on sphingolipid metabolism, especially on endogenous SM levels, and the relationship between HCV replication and endogenous SM molecular species remain to be elucidated as there are technical challenges in measuring SM levels (for both total and individual molecular species) in hepatocytes.

To address these questions, we first utilized mass spectrometry (MS)-based techniques and analyzed uninfected and HCV-infected chimeric mice harboring human hepatocytes. Second, we developed a hepatotropic SPT inhibitor, NA808, and used this tool to elucidate the effects of inhibition of sphingolipid biosynthesis on hepatocyte SM levels. Third, we tested the inhibitor's anti-HCV activity in humanized chimeric mice, and demonstrated the relationship between HCV and endogenous SM in human hepatocytes. Finally, we identified the endogenous SM molecular species carried by the DRM fraction, defining the association between these molecular species and HCV replication.

## Results

### HCV upregulates SM and ceramide levels in hepatocytes of humanized chimeric mice

**Figure 1:**
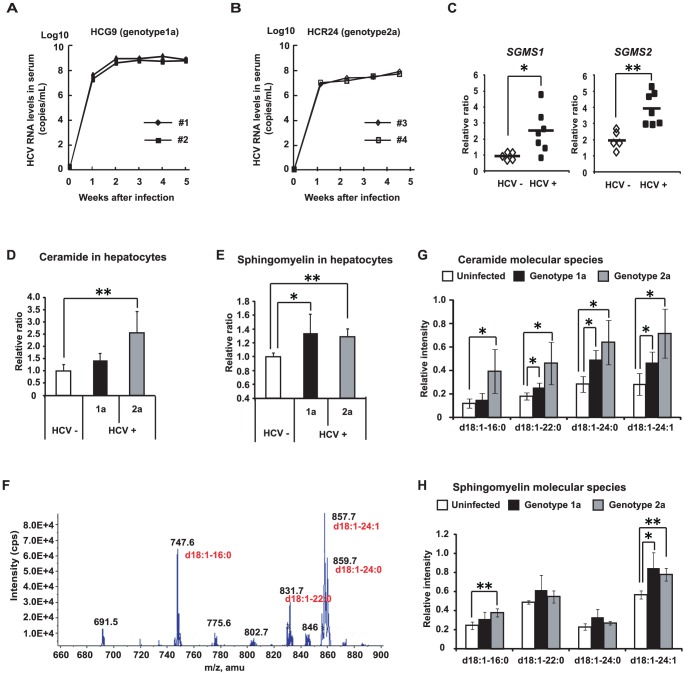
HCV alters sphingolipid metabolism. (**A, B**) Time-course studies of humanized chimeric mice inoculated with human serum samples positive for HCV genotype 1a (**A**) or 2a (**B**). (**C**) mRNA expression of *SGMS1* and *SGMS2* in uninfected (white, n = 5) and HCV genotype 1a-infected (black, n = 7) chimeric mice. (**D, E**) Effects of HCV infection on hepatocyte SM and ceramide levels in humanized chimeric mice. Relative intensity of total ceramide (**D**) and total shingomyelin (SM) (**E**) in uninfected mouse hepatocytes (white bar, n = 4), HCV genotype 1a-infected mouse hepatocytes (black bar, n = 5), and HCV genotype 2a-infected mouse hepatocytes (dark gray bar, n = 3). (**F**) Mass spectrum of SM in Bligh & Dyer extracts of a human hepatocyte cell line (HuH-7 K4). (**G, H**) Effects of HCV infection on hepatocyte SM and ceramide levels in humanized chimeric mice. Relative intensity of individual ceramide molecular species (**G**) and individual SM molecular species (**H**) in uninfected mouse hepatocytes (white bar, n = 3), HCV genotype 1a-infected mouse hepatocytes (black bar, n = 3), and HCV genotype 2a-infected mouse hepatocytes (dark gray bar, n = 3). In all cases, error bars indicate SDs. *p<0.05 and **p<0.01 compared with uninfected hepatocytes.

First, we examined the effects of HCV infection on SM biosynthesis in hepatocytes using humanized chimeric mice. The study employed a previously described mouse model (SCID/uPA) into which human hepatocytes were transplanted (see Materials and Methods). The average substitution rate of the chimeric mouse livers used in this study was over 80% [13], and HCV selectively infected human hepatocytes. This model supports long-term HCV infections at clinically relevant titers [13], [14]. Indeed, the HCV-RNA levels reached (at 4 weeks post-infection) 10^8^–10^9^ copies/mL in the genotype 1a group (**Figure 1A**) and 10^6^–10^7^ copies/mL in the genotype 2a group (**Figure 1B**).

Once serum HCV-RNA levels had plateaued, we observed elevated expression of the genes (*SGMS1* and *2*) encoding human SM synthases 1 and 2; this pattern was HCV-specific, as demonstrated by the fact that the increase was not seen in hepatitis B virus-infected mice (**Figure 1C** **and Figure S1**). SM synthases convert ceramide to SM, so we next examined SM and ceramide levels in hepatocytes of both HCV-infected and uninfected chimeric mice. SM and ceramide levels were assessed using MS spectrometry, which allows analysis of samples at the single lipid species level as well as at the whole lipidome level. MS analysis showed that the level of ceramide, the precursor to SM, was increased in hepatocytes obtained from chimeric mice infected with HCV of either genotype (**Figure 1D**). Further, MS analysis showed that infection of chimeric mice with HCG9 (genotype 1a) was associated with increased SM levels in hepatocytes (**Figure 1E**). Similarly, SM levels were elevated in the hepatocytes of HCR24 (genotype 2a)-infected chimeric mice. These results indicate that infection with HCV increases total SM and ceramide levels in human hepatocytes.

MS analysis was conducted to determine which of several molecular species of SM [15] are present in HCV-infected hepatocytes. SM molecular species were analyzed in extracts obtained from a human hepatocyte cell line (HuH-7 K4) and from hepatocytes derived from the humanized chimeric mice. We identified four major peaks as SM molecular species (*d*18∶1-16∶0, *d*18∶1-22∶0, *d*18∶1-24∶0, and *d*18∶1-24∶1), and other peaks as phosphatidylcholine (**Figure 1F**). Infection-associated increases were seen for all ceramide molecular species, with significant changes in three of four species (excepting *d*18∶1-16∶0; *p*<0.05) with genotype 1a, and in all four species with genotype 2a (*p*<0.05) (**Figure 1G**). Upon infection with HCV of either genotype, hepatocytes tended to show increased levels of all four identified SM molecular species, but the changes were significant only for one species (*d*18∶1-24∶1; *p*<0.05) in genotype 1a and for two species (*d*18∶1-16∶0 and *d*18∶1-24∶1; *p*<0.01) in genotype 2a (**Figure 1H**). In cell culture, negligible amount of SM was likely increased by HCV infection. With respect to each molecular species, d18∶1-16∶0 SM was likely increased by HCV infection (**Figure S2**). These results indicate that HCV infection increases the abundance of several SM and ceramide molecular species.

### Relationship between the SGMS genes and HCV infection

**Figure 2:**
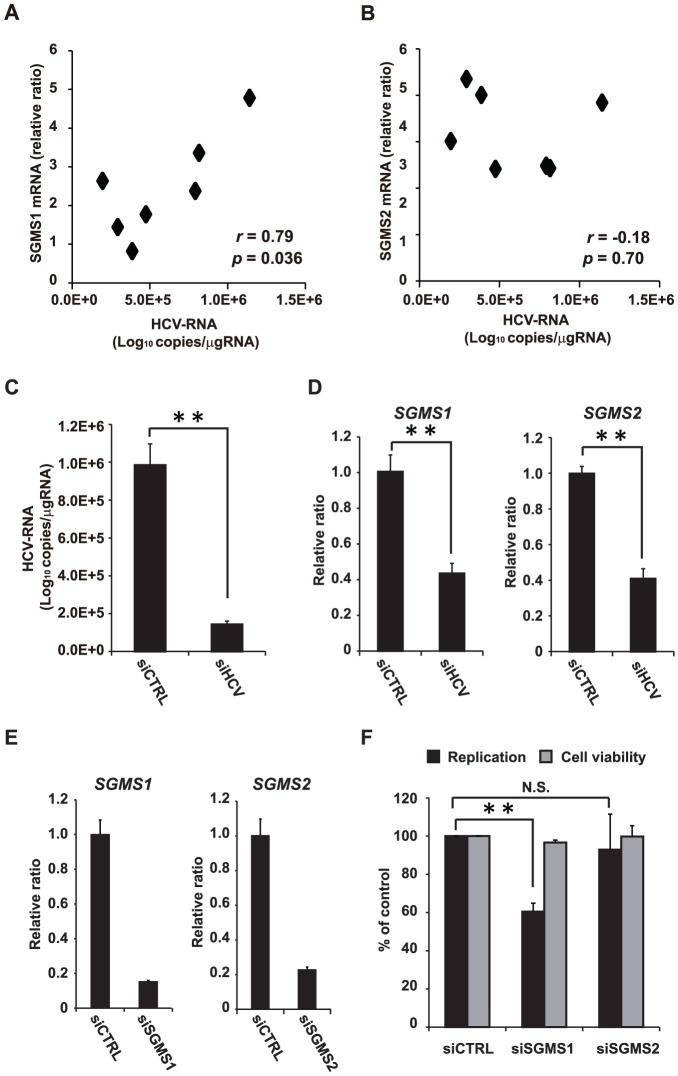
Relationship between the SGMS genes and HCV infection. (**A, B**) The correlation between SGMS1/2 and liver HCV-RNA of HCV infected humanized chimeric mice (n = 7). (**C**) The effect of silencing HCV genome RNA with siRNA (siE-R7: 1 nM) on HCV in HCV-infected cells. (**D**) The effect of silencing HCV genome RNA with siRNA (siE-R7: 1 nM) on the expression of SGMS1/2 mRNA measured by RTD-PCR. (**E**) The effect of silencing SGMS1/2 mRNA with siRNA (3 nM each) measured by RTD-PCR. (**F**) The effect of silencing SGMS1/2 mRNA with siRNA (3 nM) on HCV replication in FLR 3-1. In all cases, error bars indicate SDs. *p<0.05 and **p<0.01.

To clarify the relationship between SGMS1/2 and HCV, we investigated the correlation between SGMS1/2 expression and liver HCV-RNA in humanized chimeric mice. We found that SGMS1, but not SGMS2, had a correlation with liver HCV-RNA in HCV-infected humanized chimeric mice (**Figures 2A and 2B**).

Next, to clarify whether HCV infection of human hepatocytes increases the expression of the genes (*SGMS1* and *SGMS2*), we examined the effect of silencing HCV genome RNA on the expression of these genes in HCV-infected cells (**Figures 2C and 2D**). We found that silencing the HCV genome RNA decreases the expression of *SGMS1* and *SGMS2*.

The above results motivated us to examine the relationship between SGMS1/2 and HCV replication. Therefore, we examined the effect of SGMS1/2 mRNA silencing on HCV replication using subgenomic replicon cells [7], [16]. We observed that silencing SGMS1 mRNA suppressed HCV replication, whereas silencing SGMS2 mRNA had no such effect (**Figures 2E and 2F**). These results indicate that SGMS1 expression has a correlation with HCV replication.

### Characterization of the hepatotropic SPT inhibitor NA808

**Figure 3:**
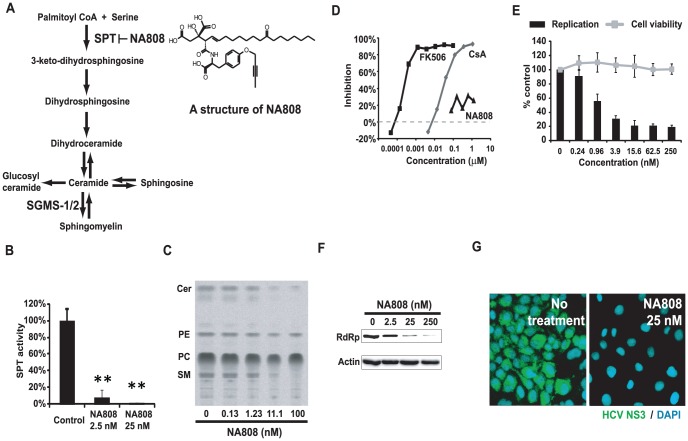
Characterization of the hepatotropic serine palmitoyltransferase inhibitor NA808. (**A**) Sphingolipid biosynthesis pathway and structure of NA808. (**B**) Activity of SPT in FLR3-1 cells after 72 h of NA808 treatment. **p<0.01 compared with control. (**C**) Results of TLC showing *de novo* sphingolipid biosynthesis in the presence of NA808. Cer = ceramide, PE = phosphatidylethanolamine, PC = phosphatidylcholine, SM = sphingomyelin. (**D**) Immunosuppressive activity of NA808. Cyclosporin A (CsA) and tacrolimus (FK-506) were used as positive controls. (**E**) Effects of NA808 on HCV replication (black bars) and cell viability (gray symbols) in FLR 3-1 replicon-containing cells. Error bars indicate SDs. (**F**) Effects of NA808 on the level of the RdRp and β-actin, as assessed by Western blotting. (**G**) Effect of NA808 on the production of HCV NS3 protein (green) in FLR3-1 replicon-containing cells, as assessed by immunofluorescence analysis. Nuclear DNA was stained with DAPI (blue).

Based on our data, we hypothesized that HCV might alter the metabolism of sphingolipids, providing a more conducive environment for progression of the viral life cycle. To explore the relationship between HCV and sphingolipids, we investigated the effect of sphingolipid biosynthesis inhibition on HCV and the lipid profiles of SM and ceramide using HCV-infected chimeric mice harboring human hepatocytes. To inhibit the biosynthesis of sphingolipids, we used NA808, a chemical derivative of NA255, which is an SPT inhibitor derived from natural compounds [7]. We found that NA808 (**Figure 3A**) suppressed both the activity of SPT (**Figure 3B**) and biosynthesis of sphingolipids (**Figure 3C**) in a dose-dependent manner.

The conventional SPT inhibitor myriocin is not clinically beneficial due to immunosuppression through restriction of T-cell proliferation [17], [18]. However, NA808 showed little immunosuppressive effect at the concentration at which NA808 suppressed HCV replication (**Figures 3D and 3E**). Moreover, pharmacokinetic analysis using [^14^C]-labeled NA808 in rat models showed that NA808 mainly accumulated in the liver and small intestine (**Table S1**). These results indicate that NA808 suppressed SPT activity, with hepatotropic and low immunosuppressive properties.

Based on these results, we then examined the effects of inhibition of sphingolipid biosynthesis with NA808 on HCV replication using subgenomic replicon cells [7], [16]. The luciferase activity of FLR3-1 showed that replication was suppressed by NA808 in a dose-dependent manner with no effect on cell viability, as measured by the WST-8 assay (**Figure 3E**). Similarly, western blot and immunofluorescence analysis showed that NA808 effectively suppressed HCV replication (**Figures 3F and 3G**).

### Inhibition of sphingolipid biosynthesis impedes HCV infection of chimeric mice

**Figure 4:**
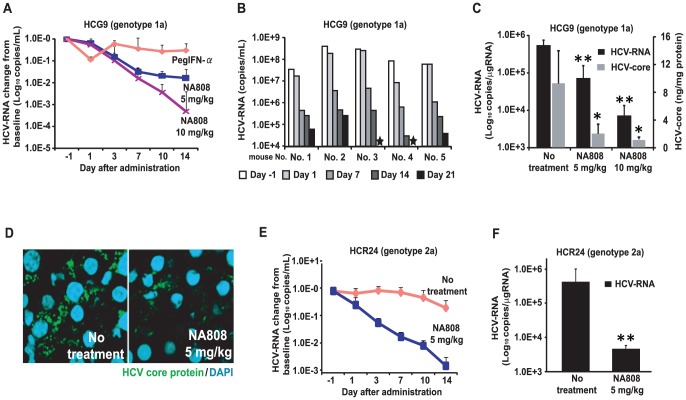
Inhibition of sphingolipid biosynthesis with hepatotropic serine palmitoyltransferase (SPT) inhibitor NA808 exerts anti-HCV effect. (**A**) Serum HCV-RNA levels in response to treatment with NA808 (blue, 5 mg/kg/day, purple, 10 mg/kg/day, n = 6 each), or PegIFN-α (pink, 30 µg/kg twice weekly, n = 4). (**B**) Effect of NA808 (5 mg/kg/day) on serum HCV-RNA levels. A star indicates that HCV-RNA was not detected. (**C**) Levels of liver HCV-RNA (black) and HCV core protein (gray) after the 14-day treatment. *p<0.05 and **p<0.01 compared with no treatment. (**D**) Histological analysis using immunofluorescent labeling of HCV core protein (green) and fluorescent staining of nuclei (blue). (**E**) Serum HCV-RNA levels in response to no treatment (pink, n = 3) or NA808 treatment (blue, 5 mg/kg/day, n = 4). (**F**) Liver HCV-RNA levels in genotype 2a-infected mice after the 14-day treatment. *p<0.05 and **p<0.01 compared with no treatment. In all cases, error bars indicate SDs.

To evaluate the effects of inhibition of sphingolipid biosynthesis in an animal model, we administered NA808 or pegylated interferon-α (PegIFN-α) via intravenous or subcutaneous injection to HCV-infected chimeric mice harboring human hepatocytes (**Table S2**). In chimeric mice infected with HCV genotype 1a, NA808 treatment led to a rapid decline in serum HCV-RNA (approximately 2–3 log units within 14 days). On the other hand, PegIFN-α produced less than a 1 log unit reduction, despite being delivered at 20 times the typical clinical dose (**Figure 4A**). Furthermore, results of 21-day NA808 treatment (5 mg/kg) in individual mice indicated that serum HCV RNA continued to decrease in all chimeric mice without viral breakthrough (**Figure 4B**). Notably, in 2 of 5 chimeric mice, serum HCV-RNA was not detectable at the end of the 21-day regimen. Consistent with this observation, the levels of both hepatic HCV-RNA and HCV core protein decreased significantly (*p*<0.01 and *p*<0.05, respectively) following NA808 treatment, these effects being dose dependent (**Figure 4C**). Immunofluorescence analysis and immunohistochemistry confirmed the reduced abundance of HCV core protein after 14 days of treatment (**Figure 4D** **and Figure S3**).

In genotype 2a-infected chimeric mice, NA808 decreased serum HCV-RNA by approximately 3 log units within 14 days (**Figure 4E**). NA808-treated mice displayed a corresponding reduction in hepatic HCV-RNA (**Figure 4F**). NA808 did not affect body weight or human serum albumin levels (**Figures S4A and S4B**). Furthermore, hematoxylin and eosin (H&E) staining revealed little morphological change in response to treatment with NA808. Immunofluorescence analysis also indicated that NA808 did not affect the production of human albumin (**Figure S4C**). Thus, inhibition of sphingolipid biosynthesis by an SPT inhibitor impeded HCV replication in an animal infection model, regardless of HCV genotype.

### Inhibition of SPT decreases ceramide and SM levels in hepatocytes of humanized chimeric mice

We next investigated the effects of sphingolipid biosynthesis inhibition on SM and ceramide levels in hepatocytes of humanized chimeric mice. Pharmacokinetic analysis in a rat model indicated that NA808 has hepatotropic properties (**Table S1**). Consistent with this analysis, our study in chimeric mice also indicated that the NA808 concentration was much higher in the liver than in serum (**Figure S5**). Furthermore, we observed that serum SM content was not decreased by NA808 treatment (**Figure S6**), in contrast to the effects previously observed for myriocin, another SPT inhibitor [19].

**Figure 5:**
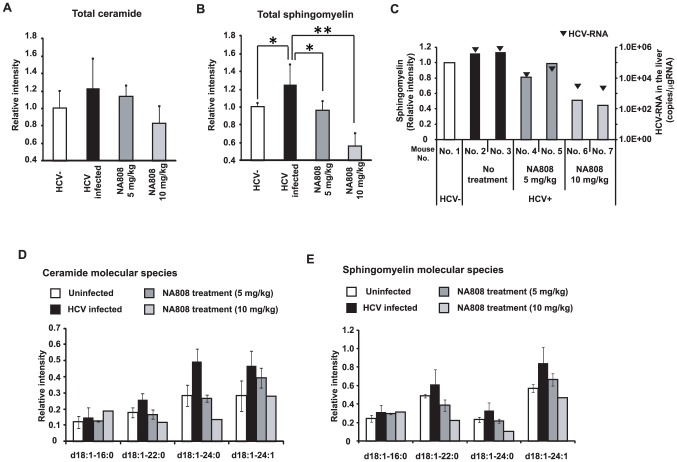
Effects of NA808 treatment on sphingomyelin (SM) and ceramide (total and individual molecular species). (**A, B**) Relative ratio of total ceramide (**A**) and SM (**B**) in uninfected mice (white, n = 4), HCV genotype 1a-infected mice (black, n = 5), and HCV-infected mice treated with NA808 for 14 days (dark gray, 5 mg/kg, n = 4; light gray, 10 mg/kg, n = 3). *p<0.05 and **p<0.01 compared with HCV-infected mice. (**C**) SM levels (bars) and HCV RNA levels (black arrowhead) in the livers of mice treated for 14 days with NA808 (5 or 10 mg/kg/day) and untreated chimeric mice. (**D, E**) Relative intensities of individual ceramide molecular species (**D**) and individual SM molecular species (**E**) in uninfected mice (white, n = 3), HCV-infected mice (black, n = 3), and HCV-infected mice treated with NA808 for 14 days (dark gray, 5 mg/kg, n = 2; light gray, 10 mg/kg, n = 1). In all cases, error bars indicate SDs.

In HCV-infected chimeric mouse hepatocytes, MS analysis indicated that HCV infection resulted in increased ceramide and SM levels. However, treatment of infected animals with NA808 (5 mg/kg) attenuated this increase in ceramide and SM levels in hepatocytes, and the change in SM was significant (*p*<0.05) compared to the level observed in HCV-infected chimeric mice with no treatment. This effect of NA808 on ceramide and SM levels was dose-dependent (**Figures 5A and 5B**). We also found that SM levels and hepatic HCV-RNA were correlated (**Figure 5C**).

Interestingly, treatment with NA808 effectively decreased two specific SM and ceramide molecular species (*d*18∶1-22∶0 and *d*18∶1-24∶0), slightly decreased one other species (*d*18∶1-24∶1), and hardly decreased another (*d*18∶1-16∶0). Further, we found that among SM and ceramide molecular species, *d*18∶1-16∶0 did not change (**Figures 5D and 5E**). These results indicate that the effects of sphingolipid biosynthesis inhibition varied among the molecular species.

Considering these results, we found a discrepancy in SM molecular species which were considered to be important for HCV replication. To elucidate the relationship between SM molecular species and HCV replication, we attempted to identify endogenous SM molecular species comprising the DRM fraction and to evaluate the effects of HCV infection and inhibition of sphingolipid biosynthesis on SM levels of the DRM.

### Relationship between endogenous SM molecular species constituting the DRM and HCV replication

**Figure 6:**
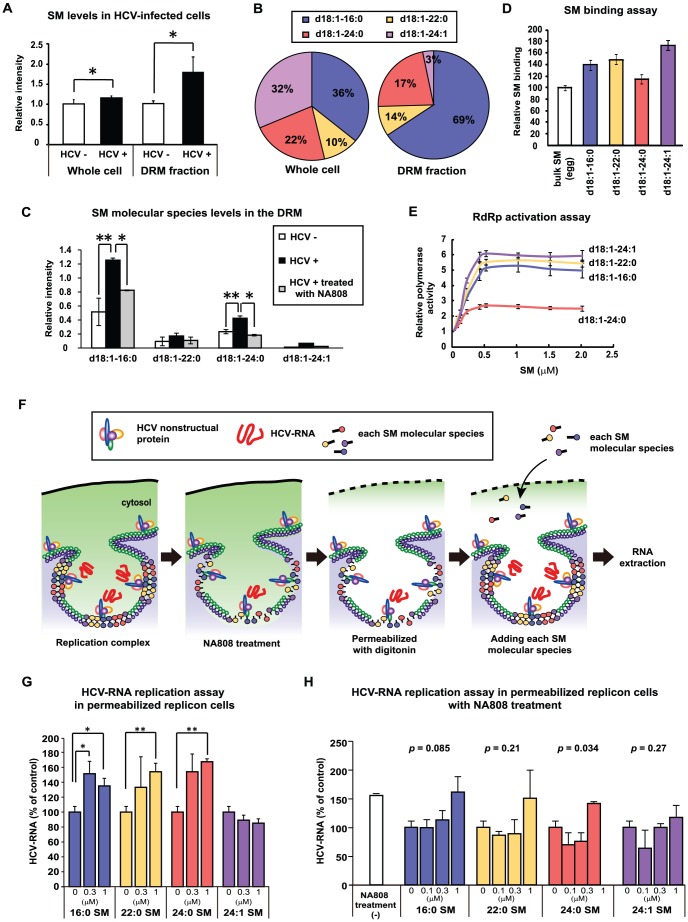
Specific sphingomyelin molecular species upregulated by HCV promote HCV replication on the detergent-resistant membrane fraction. (**A**) Comparison of the relative amounts of SM, as measured by MS analysis, in whole cells and the DRM fraction of mock-infected (HuH-7 K4 cells) (white, n = 6; whole cells, n = 3; DRM fraction) and HCV (JFH-1)-infected cells (JFH/K4 cells) (black, n = 6; whole cells, n = 3; DRM fraction). (**B**) Composition ratio of SM molecular species in whole cells and DRM fraction of HCV-infected cells. (**C**) Relative intensities of each SM molecular species in the DRM fraction of mock-infected cells (white, n = 2) and HCV-producing cells without (black, n = 2) or with NA808 treatment (gray, n = 2). (**D**) Results of the ELISA SM binding assay (n = 3 each). (**E**) Average activation kinetics of each SM molecular species on HCR6 (genotype 1b) RdRp (n = 3 each). (**F**) Scheme of HCV-RNA replicase assay using digitonin-permeabilized cells. (**G, H**) Effect of each SM molecular species on HCV-RNA in digitonin-permeabilized replicon cells treated without (G) or with 10 nM NA808 (H) (n = 3 each). In all cases, error bars indicate SDs. *p<0.05 and **p<0.01.

We previously reported that SM interacts with RdRp, allowing it to localize to the DRM fraction where HCV replicates and activates RdRp [7], [8], and that suppression of SM biosynthesis disrupts the association between RdRp and SM in the DRM fraction, resulting in suppression of HCV replication [7], [8]. In the present study, treatment with NA808 decreased SM levels in the DRM fraction; the decreased presence of SM correlated with decreased RdRp abundance, but the same effect was not seen for HCV nonstructural protein 3 (**Figures S7A–C**). Given these results, we investigated whether HCV replication was induced by elevated SM levels. Specifically, we compared SM levels in the DRM fraction between HCV-infected hepatocytes and uninfected hepatocytes. MS analysis showed that HCV increased SM levels in the DRM fraction more remarkably than in whole cells (**Figure 6A**). Next, we identified SM molecular species composing the DRM fraction and found that the composition ratio of SM molecular species was distinct between whole cells and DRM fractions in both HCV-infected and uninfected hepatocytes (**Figure 6B** **and Figure S8**). The DRM was composed primarily (69%) of *d*18∶1-16∶0, followed (in decreasing order) by *d*18∶1-24∶0, *d*18∶1-22∶0, and *d*18∶1-24∶1; the abundance of all SM molecular species increased upon HCV infection (**Figure 6C**). Further, NA808 treatment decreased all SM molecular species in the DRM fraction. Consistently, NS3 protease inhibitor decreased all SM molecular species in the DRM fraction of subgenomic replicon cells (**Figure S9**).

To address the association between RdRp and the endogenous SM molecular species composing the DRM, we used high-performance liquid chromatography (HPLC) to separate each SM molecular species from bulk SM derived from bovine milk and brain. We evaluated the relationship between RdRp and these endogenous SM molecular species using *in vitro* analysis. Enzyme-linked immunosorbent assay (ELISA) indicated that these endogenous SM molecular species bound to RdRp more readily than the bulk SM derived from milk as a positive control (**Figure 6D**). Further, *in vitro* HCV transcription analysis showed that three SM species (*d*18∶1-16∶0, *d*18∶1-22∶0, and *d*18∶1-24∶1) increased *in vitro* RdRp activation by approximately 5-fold, whereas the *d*18∶1-24∶0 species increased activation by 2-fold (**Figure 6E**). In a previous study, the soluble RdRp without its C-terminal hydrophobic 21-amino-acid sequence was used in *in vitro* analysis [8], and whether the relationship between RdRp and SM proved in this analysis reflected the state in the membranous replication complex remains to be elucidated. Therefore, we attempted to examine the effect of endogenous SM molecular species on HCV replicase activity *in vivo* using digitonin-permeabilized semi-intact replicon cells, which permit monitoring of the function of the active HCV replication complex (**Figure 6F**) [20]. This *in vivo* analysis also enabled us to deliver the extrinsically added SM molecular species directly to the cytosol. This RNA replication assay indicated that the endogenous SM molecular species (*d*18∶1-16∶0 and *d*18∶1-24∶0) enhanced HCV-RNA replication, these species being consistent with the two SM molecular species that primarily constitute the DRM and are decreased significantly by NA808 treatment (**Figures 6G and 6H**). These results suggest that HCV infection modifies the levels of specific endogenous SM molecular species, which in turn enhance HCV-RNA replication by interacting with RdRp.

## Discussion

In this study, we showed that HCV alters sphingolipid metabolism, resulting in a better environment for viral replication. Specifically, HCV increased SM content in the DRM fraction; this step is essential for viral replication since SM is a key component of the membranous replication complex and interacts with RdRp. Employing MS analysis, we identified endogenous SM molecular species (located in the DRM fraction) that increased upon HCV infection, and demonstrated that these endogenous SM molecular species interact directly with RdRp, enhancing HCV replication. Thus, we concluded that HCV modulates sphingolipid metabolism to promote viral replication.

We found that the expression levels of SGMS1/2 and the content of SM and ceramide in HCV-infected humanized chimeric mouse livers was increased (Figure 1). Our measurement revealed that chronic HCV infection promoted sphingolipid biosynthesis. HCV is known to induce cellular stress [21], [22]. A variety of cell stressors increase intracellular ceramide content during the execution phase of apoptosis [23], [24], indicating that ceramide is a proapoptotic lipid mediator. Furthermore, activation of ceramide-metabolizing enzymes such as glucosylceramide synthase and SM synthase can attenuate apoptosis by decreasing the intracellular ceramide content [25], [26]. We found that HCV infection correlated with increased mRNA levels of the genes that encode human SM synthases (*SGMS1/2*) and glucosylceramide synthase (*UGCG*) (data not shown). Thus, the increase in ceramide levels observed in our study was likely to activate enzymes that transfer ceramide to other sphingolipids. On the other hand, Diamond et al. reported on lipidomic profiling performed over the time course of acute HCV infection in cultured Huh-7.5 cells and observed that specific SM molecular species were decreased 72 h after HCV infection [27]. Given that their study focused on acute HCV infection, the reason for this discrepancy may be due to the severity of infection, suggesting that the influence of HCV infection on sphingolipid metabolism differs between acute and chronic infections. We also demonstrated that HCV infection correlates with increased abundance of specific SM and ceramide molecular species, with the profiles of individual lipids differing for infection by HCG9 (genotype 1a) and HCR24 (genotype 2a). The precise mechanism and meaning of these differences remain to be elucidated.

Our results indicated that SGMS1 expression had a correlation with HCV replication. This indicates that SM synthesized by SGMS1 contributes to HCV replication. A previous report revealed that in cultured cell lines, SGMS1 localizes in Golgi apparatus while SGMS2 localizes in the plasma membrane [28]. Thus, the results of this previous report suggest that SMs synthesized by SGMS1 can be easily incorporated into membranous replication complexes. As for SGMS2, we found that HCV infection significantly increased the expression of SGMS2, although the relationship between SGMS2 and HCV replication was hardly seen in this study. The relationship between SGMS2 and HCV propagation, thus, is an issue that should be elucidated in future studies.

We also demonstrated in this study that reduction of SM molecular species by NA808, a hepatotropic SPT inhibitor with little immunosuppressive activity, inhibits HCV replication in humanized chimeric mice regardless of viral genotype (Figure 4). Notably, treatment with NA808 (5 mg/kg) restored SM and ceramide levels in the liver to the levels observed in uninfected chimeric mice (Figure 5). Apparently, a slight reduction in SM had a significant influence on HCV, indicating that SM plays an important role in the HCV life cycle. SM is required for many viral processes in host-pathogen interactions [29]–[31]. For instance, viral envelopes of human immunodeficiency virus type 1 (HIV-1) and herpes simplex virus (HSV) are enriched with SM, which is necessary for efficient virus infectivity [32], [33]. With regard to HCV, in addition to efficient virus infectivity [34], SM is present in the raft domain, which serves as a site of virus replication, together with other sphingolipids and cholesterol [6]. Moreover, SM is a component of VLDL whose assembly component and pathway is required for HCV morphogenesis and secretion [34], [35]. The above-mentioned observations suggest that SM plays a multifaceted role in the HCV life cycle; therefore, SM is likely to be a good therapeutic target.

HCV is thought to replicate in a specialized compartment characterized as a DRM (designated as the membranous replication complex) [6]. SM, cholesterol, and phosphatidylinositol (PI) are thought to be the lipids that make up the membranous replication complex. With regard to PI, several siRNA screening have recently identified type III phosphatidylinositol 4-kinases (PI4K) as crucial host factors for HCV replication [36]–[39]. In HCV replicon containing cells, PI4P distribution is altered and enriched in the membranous replication complex by PI4KIIIα synthesis. Although the ability of PI to influence membrane bending and regulate intracellular processes (e.g. vesicle fusion, budding, and sorting) has been reported, the role of PI4P in the formation of the membranous replication complex remains to be elucidated. SM and cholesterol organize the solid membrane characterized as the DRM, where HCV replicates [6]. In fact, we and other groups demonstrated that reduction of SM and cholesterol suppressed HCV replication [7], [9], [12], [40]. We performed the immunofluorescent analysis using lysenin. However, lysenin did not co-localize with NS4B protein. To date, it has been reported that lysenin-binding to SM is increased in the form of SM clusters, and that glycosphingolipids hinder lysein-binding to SM [41] Lipid rafts form of HCV replication complex do not have the characters of lysenin-binding to SM.

Further, the role of SM is not only to act as a constituent of the membranous replication complex, but also to bind and activate RdRp [7], [8]. In this study, to gain further insight into the HCV membranous replication complex, we attempted to analyze which SM molecular species comprise the membranous replication complex, given that the diversity of molecular species is believed to be responsible for the physiochemical properties of the biomembrane [42] (Figure 6). We found that the composition ratio of SM molecular species observed in this study was quite different between the whole cell and DRM fractions. Further, to identify whether these SM molecular species contribute to HCV replication, we conducted rescue experiments using HCV replicon-containing cells (carrying intact RdRp and active membranous replication complexes) in which each SM molecular species was extrinsically added to replicon cells treated with NA808. However, in this experiment, addition of SM caused cell death. Therefore, we used digitonin-permeabilized semi-intact replicon cells, which enabled us to deliver the extrinsically added SM molecular species directly to the cytosol without catalytic effect and permitted monitoring of intact RdRp and replication complexes. We demonstrated that the specific endogenous SM molecular species (*d*18∶1-16∶0 and *d*18∶1-24∶0) enhance HCV-RNA replication, these species being consistent with the two SM molecular species which mainly constitute the DRM. Collectively, these results suggest that the HCV replication complex characterized as DRM is the specialized compartment that is composed of SM molecular species. These findings will provide new insights into the formation of the HCV replication complex and the involvement of host lipids in the HCV life cycle.

## Materials and Methods

### Ethics statement

This study was carried out in strict accordance with both the Guidelines for Animal Experimentation of the Japanese Association for Laboratory Animal Science and the recommendations in the Guide for the Care and Use of Laboratory Animals of the National Institutes of Health. All protocols were approved by the ethics committee of Tokyo Metropolitan Institute of Medical Science. The patient with HCV infection who provided the serum samples gave written informed consent before blood collection.

### Cells

The HCV subgenomic replicon cells FLR3-1 (genotype 1b, Con-1) was cultured at 37°C in Dulbecco's modified Eagle's medium GlutaMax-I (Invitrogen, Carlsbad, CA, USA) supplemented with 10% fetal bovine serum (FBS) and 0.5 mg/mL G418. HuH-7 K4 cells (cured of HCV by IFN treatment) and the JFH/K4 cells persistently infected with the HCV JFH-1 strain were maintained in DMEM containing 10% FCS and 0.1 mg/mL penicillin and streptomycin sulfate. MH-14 cells were grown in Dulbecco's modified Eagle's medium supplemented with 10% fetal bovine serum, 100 U/mL nonessential amino acids, 0.1 mg/mL penicillin and streptomycin sulfate, and 0.5 mg/mL G418.

### siRNA assay

siCONTROL, siSGMS1, and siSGMS2 were purchased from Dharmacon RNA Technologies (Lafayette, CO, USA). The siCONTROL Non-Targeting siRNA #3 was used as the negative control siRNA. We used siRNAs against the HCV genome (siE-R7) [16]. The chemically synthesized siRNAs were transfected into cells using Lipofectamine RNAiMAX (Invitrogen) and Opti-MEM (Invitrogen) by reverse-transfection. Cells were characterized at 96 h after transfection.

### Serine palmitoyltransferase activity

We assessed SPT activity in the liver as previously described, with minor modifications [43]. Briefly, frozen cells were homogenized in HEPES buffer (10 mM HEPES, 2 mM sucrose monolaurate, and 0.25 M sucrose, pH 7.4), and homogenates were centrifuged at 10,000×*g* for 20 min. From the resulting supernatant, samples containing 200 µg protein were assayed for SPT activity using [^14^C]-serine and palmitoyl-CoA (Sigma-Aldrich, St. Louis, MO, USA) as substrates.

### Proliferation assay

Human peripheral blood cells (AllCells, Emeryville, CA, USA) were plated onto 96-well plates and treated with phytohemagglutinin with or without immunosuppressant reagents. After 2 days of stimulation, [^3^H]-thymidine-containing growth medium was added, and the cultures were incubated for another 18 h. T-cell proliferation was assessed by comparing the level of thymidine incorporation to that in the stimulated control.

### Anti-hepatitis C virus assay in Huh-7 cells harboring subgenomic replicons

Replication was determined after 72 h with a Bright-Glo luciferase assay kit (Promega, Madison, WI, USA). The viability of replicon cells was determined using a cell counting kit (Dojindo, Kumamoto, Japan) according to the manufacturer's instructions.

### Western blot analysis

Cells were resuspended in lysis buffer (10 mM Tris, pH 7.4 containing 1% SDS, 0.5% Nonidet P-40, 150 mM NaCl, 0.5 mM EDTA, and 1 mM dithiothreitol). Ten micrograms of the resulting protein sample were electrophoresed on a 10% sodium dodecyl sulfate-polyacrylamide gel and subsequently transferred to a polyvinylidene difluoride membrane (Immobilon-P; Millipore, Billerica, MA, USA). HCV nonstructural protein 3 (NS3) and nonstructural 5B polymerase (RdRp) were detected with rabbit anti-NS3 polyclonal antibody (R212) and mouse anti-RdRp monoclonal antibody (5B-14) prepared in our laboratory. β-Actin was detected with anti-β-actin monoclonal antibody (Sigma-Aldrich).

### Immunofluorescent staining of hepatitis C virus replicon cells

After treatment with 25 nM NA808 for 96 h, FLR3-1 cells were probed with anti-NS3 polyclonal antibody (R212; the primary antibody). Next, an anti-rabbit IgG-Alexa 488 conjugate (Invitrogen) was applied as the secondary antibody.

### Thin-layer chromatography analysis

Thin-layer chromatography (TLC) analysis was performed as described previously [9]. Briefly, cells were incubated with [^14^C]-serine in Opti-MEM (Invitrogen). Cells extracts were obtained using the Bligh & Dyer method [44] and were spotted onto Silica Gel 60 TLC plates (Merck, Darmstadt, Germany) for separation. Radioactive spots were detected using a BAS 2000 system (Fuji Film, Kanagawa, Japan).

### Membrane flotation assay

Cells were lysed in TNE buffer (25 mM Tris-HCl, 150 mM NaCl, 1 mM EDTA) and passed 20 times through a 25-gauge needle. Nuclei and unbroken cells were removed by centrifugation at 1,000×*g* for 5 min. After ensuring that the amount of total protein was equivalent across all samples, cell lysates were treated with 1% Triton on ice for 30 min and then subjected to a sucrose gradient (10%, 30%, and 40%). The sucrose gradient was centrifuged at 247,220×*g* in a Beckman SW41 Ti rotor (Beckman Coulter Inc., Brea, CA, USA) for 14 h at 4°C. Fractions (1 mL) were collected from the top of the gradient.

### Infection of mice with hepatitis C virus genotypes 1a and 2a

Chimeric mice infected with HCV were prepared as previously described [45]. Briefly, approximately 40 days after the transplantation procedure, mice were intravenously injected with 5×10^5^ copies/mouse of HCG9 (genotype 1a) or HCR24 (genotype 2a) that had been collected from patient serum.

### Quantification of HCV RNA by real-time polymerase chain reaction

Total RNA was purified from 1 µL of chimeric mouse serum using SepaGene RV-R (Sanko Junyaku Co. Ltd., Tokyo, Japan) and from liver tissue using Isogene (Nippon Gene Co. Ltd., Tokyo, Japan). HCV RNA was quantified by quantitative real-time polymerase chain reaction (PCR) using previously reported techniques [9]. For serum, this technique has a lower limit of detection of 4000 copies/mL. Therefore, samples in which HCV RNA was undetectable were assigned this minimum value.

### Quantification of HCV core protein by ELISA

Liver specimens were homogenized in TNE buffer. Aliquots of 5 µg of total protein were assayed for core protein levels with an Ortho HCV core protein ELISA kit (Eiken Chemical, Tokyo, Japan).

### Indirect immunofluorescence analysis

The primary antibody for immunofluorescence analysis of liver sections was anti-HCV core protein monoclonal antibody (5E3) [46]. Monoclonal antibody labeling was followed by staining with anti-mouse IgG Alexa-488. The nuclei were stained using 4′,6-diamidino-2-phenylindole (DAPI).

### Gene expression analysis

To measure mRNA levels, total RNA samples were extracted from the mouse livers and cDNA was synthesized using a High-Capacity cDNA Reverse Transcription Kit (Applied Biosystems, Foster City, CA, USA). The cDNA solution was assessed by quantitative PCR performed with TaqMan Gene Expression Assays (Applied Biosystems) and an ABI 7700 Sequence Detection System (Applied Biosystems).

### Quantification of SM and ceramide in liver

We quantified liver SM and ceramide levels using a mass spectrometer (MS). Electrospray ionization (ESI)-MS analysis was performed using a 4000Q TRAP quadrupole-linear ion trap hybrid MS (AB SCIEX, Foster City, CA, USA) with an UltiMate 3000 nano/cap/micro-liquid chromatography system (Dionex Corporation, Sunnyvale, CA, USA) combined with an HTS PAL autosampler (CTC Analytics AG, Zwingen, Switzerland). The total lipid fractions expected to contain SM and ceramide, were subjected directly to flow injection and were selectively analyzed by neutral loss scanning of 60 Da (HCO_2_+CH_3_) from SM [M+HCOO]^−^ in the negative ion mode, and multiple-reaction monitoring using a combination of ceramide [Cer−H_2_O+H]^+^ and the product (long-chain base) [LCB−H_2_O+H]^+^ in the positive ion mode [47], [48]. The mobile phase composition was acetonitrile∶methanol∶water at 6∶7∶2 (0.1% ammonium formate, pH 6.8) and a flow rate of 10 µL/min. The typical injection volume was 3 µL of total lipids, normalized by protein content.

LC/ESI-MS analysis was performed using quadrupole/time of flight (Q-TOF) micro with an ACQUITY UPLC system (Waters Corporation, Milford, MA, USA) in the negative ion mode and an Agilent 6230 with an Agilent 1290 Infinity LC system (Agilent Technologies, Inc., Loveland, CO, USA) in the positive ion mode. Reversed-phase LC separation was achieved using an ACQUITY UPLC BEH column (150 mm×1.0 mm i.d., Waters Corporation) at 45°C. The mobile phase was acetonitrile∶methanol∶water at 19∶19∶2 (0.1% formic acid+0.028% ammonia) (A) and isopropanol (0.1% formic acid+0.028% ammonia) (B), and the composition was produced by mixing these solvents. The gradient consisted of holding A∶B at 90∶10 for 7.5 min, then linearly converting to A∶B at 70∶30 for 32.5 min, and then linearly converting to A∶B at 40∶60 for 50 min. The detailed procedure for LC/ESI-MS was described previously [49], [50].

### Separation of SM molecular species by HPLC

Bovine milk or brain SM (Avanti Polar Lipids. Inc., Alabaster, AL, USA) was dissolved in chloroform∶methanol (2∶1), then separated according to molecular species by reversed-phase HPLC. The *d*18∶1-16∶0, 22∶0, and 24∶0 molecular species of SM were isolated from bovine milk SM, while the *d*18∶1-24∶0 and 24∶1 molecular species were isolated from brain SM. Bovine milk and brain SM were then separated on Senshu PAK ODS (C18) columns (Senshu Scientific Co., Ltd., Tokyo, Japan) using methanol as the eluting solvent at a flow rate of 1 mL/min. The fatty acid compositions of the purified fractions were analyzed by LC/ESI-MS. The amount of SM in each fraction was quantified using an SM assay kit (Cayman Chemical, Ann Arbor, MI, USA). We confirmed that the purity of each molecular species was approximately 90% without *d*18∶1-24∶1 (about 70%) (data not shown).

### *In vitro* HCV transcription

*In vitro* HCV transcription was performed as previously described [8].

### SM binding assay using ELISA

An SM binding assay was performed as previously described [8] using rabbit anti-HCV RdRp sera (1∶5000) and an HRP-conjugated anti-rabbit IgG antibody (1∶5000). Optical density at 450 nm (OD_450_) was measured on a Spectra Max 190 spectrophotometer (Molecular Devices, Sunnyvale, CA, USA) using the TMB Liquid Substrate System (Sigma).

### RNA replication assays in permeabilized replicon cells

The analysis using digitonin-permeabilized replicon cells was performed as previously described [20] with minor modifications. Briefly, MH-14 cells of about 80% confluency were pre-cultured for 2 h in complete Dulbecco's modified Eagle's medium containing 5 µg/mL actinomycin D (Nacalai Tesque, Kyoto, Japan), then washed with cold buffer B (20 mM HEPES-KOH (pH 7.7 at 27°C), 110 mM potassium acetate, 2 mM magnesium acetate, 1 mM EGTA, and 2 mM dithiothreitol). The cells were permeabilized by incubation in buffer B containing 50 µg/mL digitonin for 5 min at 27°C, and the reaction was stopped by washing twice with cold buffer B. The permeabilized cells were then incubated for 4 h at 27°C in the reaction mixture with or without each lipid. The reaction mixture consisted of 2 mM manganese(II) chloride, 1 mg/mL acetylated bovine serum albumin (Nacalai Tesque), 5 mM phosphocreatine (Sigma), 20 units/mL creatine phosphokinase (Sigma), 50 µg/mL actinomycin D, and 500 µM each of ATP, CTP, GTP, and UTP (Roche Diagnostics, Basel, Switzerland) in buffer B (pH 7.7). Total RNA was purified by the acid guanidinium-phenol-chloroform method. In this assay, considering that the estimated SM content in human hepatocytes is 3–4 nmol/mg protein, as demonstrated by MS analysis (**Figure S10**), the amount of SM we added in the replicase assay was 0.3–1 µM. (i.e. 0.03–0.3 nmol/0.3 mL/0.1 mg protein/12 well; the reaction volume in the replicase assay was 0.3 mL/12 wells and each well of the 12 well cell culture plates contained approximately 0.1 mg protein.)

### Statistical analysis

Statistical analysis was performed using the Student's *t*-test equipped with Excel 2008 (Microsoft, Redmond, WA, USA). To measure the strength of the association, Pearson correlation coefficient was calculated using Excel 2008. A *p*-value<0.05 was considered statistically significant.

## Supporting Information

Figure S1 **Impacts of HBV infection on expression of sphingomyelin (SM) biosynthesis genes.** mRNA expression of *SGMS1* and *SGMS2* genes (encoding SM synthases 1 and 2, respectively) in uninfected (white) and infected (black) chimeric mice (n = 5 per group).
(JPG)

Figure S2 **Effect of HCV infection in cultured cells.** Comparison of the relative amounts of SM, as measured by MS analysis, in mock-infected (HuH-7 K4 cells) (white) and HCV (JFH-1)-infected cells (JFH/K4 cells) (black) (n = 1 per group).
(JPG)

Figure S3 **The expression of HCV core protein in HCV-infected chimeric mice.** Histological analysis using immunohistochemical labeling of HCV core protein.
(JPG)

Figure S4 **Effects of NA808 on HCV-infected chimeric mice.** (**A**) Average body weight of mice during treatment. (**B**) Average human albumin concentrations in the sera of mice during treatment. (**C**) Histological analysis using H&E staining and immunofluorescent labeling of human albumin (red). In all cases, error bars indicate SDs.
(JPG)

Figure S5 **Concentrations of NA808 in chimeric mice receiving NA808 treatment.** Concentration of NA808 in the liver (gray) and serum (black) of chimeric mice treated with 5 mg/kg or 10 mg/kg NA808. Stars indicate that NA808 level was not detected.
(JPG)

Figure S6 **Sphingomyelin (SM) levels in the serum of chimeric mice receiving NA808 treatment.** SM levels in the serum of chimeric mice (n = 3 per group) that were uninfected (HCV−), or infected (HCV+) but untreated or treated with 5 or 10 mg/kg NA808. Error bars indicate SDs.
(JPG)

Figure S7 **Effects of NA808 on associations between the HCV nonstructural 5B polymerase (RdRp) and sphingomyelin (SM).** (**A**) Comparison of SDS-PAGE and TLC results for replicon cells receiving no treatment (Control) or NA808 treatment (NA808). NA808 dosage was 2.5 nM (for TLC) or 25 nM (for SDS-PAGE). (**B**) Relative band intensities of RdRp and NS3 in detergent-resistant membrane (DRM) fractions from cells receiving no treatment (Control) or 25 nM NA808 treatment (NA808). (**C**) Relative band intensities of SM in DRM fractions from cells receiving no treatment (Control) or 2.5 nM NA808 treatment (NA808).
(JPG)

Figure S8 **Composition ratio of SM molecular species in whole cells and DRM fraction of uninfected cells.**
(JPG)

Figure S9 **Effect of NS3 protease inhibitor on SM molecular species in the DRM fractions of subgenomic replicon cells.** (A) Effect of NS3 protease inhibitor (VX950) on HCV replication (dark grey bars) and cell viability (light grey bars) in FLR3-1 replicon-containing cells. Error bars indicate SD. (B) Effect of NS3 protease inhibitor (VX950; 3 µM) on SM molecular species of DRM fractions of FLR 3-1 replicon-containing cells. Error bars indicate SDs.
(JPG)

Figure S10 **The estimated SM content in human hepatocytes.** Left bar (white) indicates the intensity of SM internal standard (SM d18∶0-12∶0; 1 nmol) by mass spectrometer. Right bar indicates the intensity of 1 mg protein of human hepatocyte (HuH-7 K4).
(JPG)

Table S1 **Distribution of radioactivity in tissues after a single intravenous administration of [^14^C] NA808 at 2 mg/kg to non-fasting male rats.**
(PDF)

Table S2 **Treatment administration for HCV-infected chimeric mice.** Administration of reagents was started at day 0. The amount of NA808 was adjusted according to the body weight of the mice. Dose began at 5 mg/kg or 10 mg/kg and was reduced by half at each 10% reduction in body weight (half circle). At 20% reduction, administration was discontinued. Open circle indicates each manipulation was performed as required.
(PDF)

Text S1 **Materials and methods for supporting information.** Methods for “Infection of chimeric mice with hepatitis B virus”, “Quantification of human albumin”, “Histological staining and indirect immunofluorescence analysis”, and “Quantification of sphingomyelin (SM) in serum” are described.
(DOCX)
