# Supplementary figures and images for: Self-Enhancement of Hepatitis C Virus Replication by Promotion of Specific Sphingolipid Biosynthesis
Source: PLoS Pathog. 2012 Aug 16;8(8):e1002860. doi: 10.1371/journal.ppat.1002860 (PMC3420934; doi:10.1371/journal.ppat.1002860)

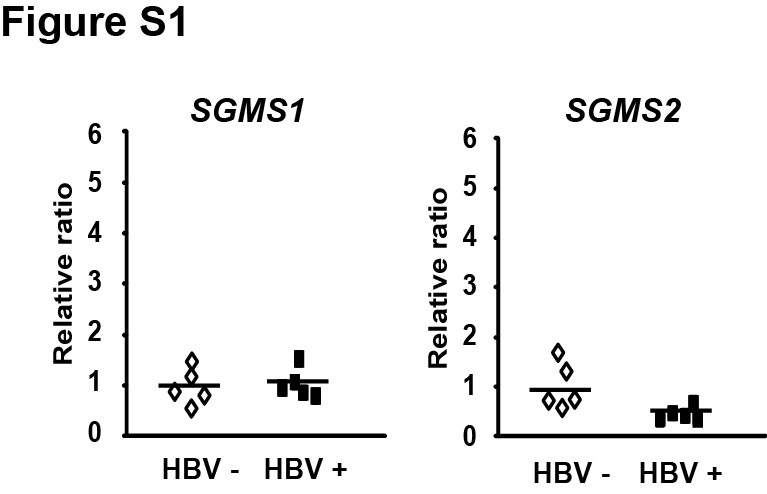

Supplement: Figure S1 — Impacts of HBV infection on expression of sphingomyelin (SM) biosynthesis genes. mRNA expression of SGMS1 and SGMS2 genes (encoding SM synthases 1 and 2, respectively) in uninfected (white) and infected (black) chimeric mice (n = 5 per group). (JPG) [file ppat.1002860.s001.jpg]

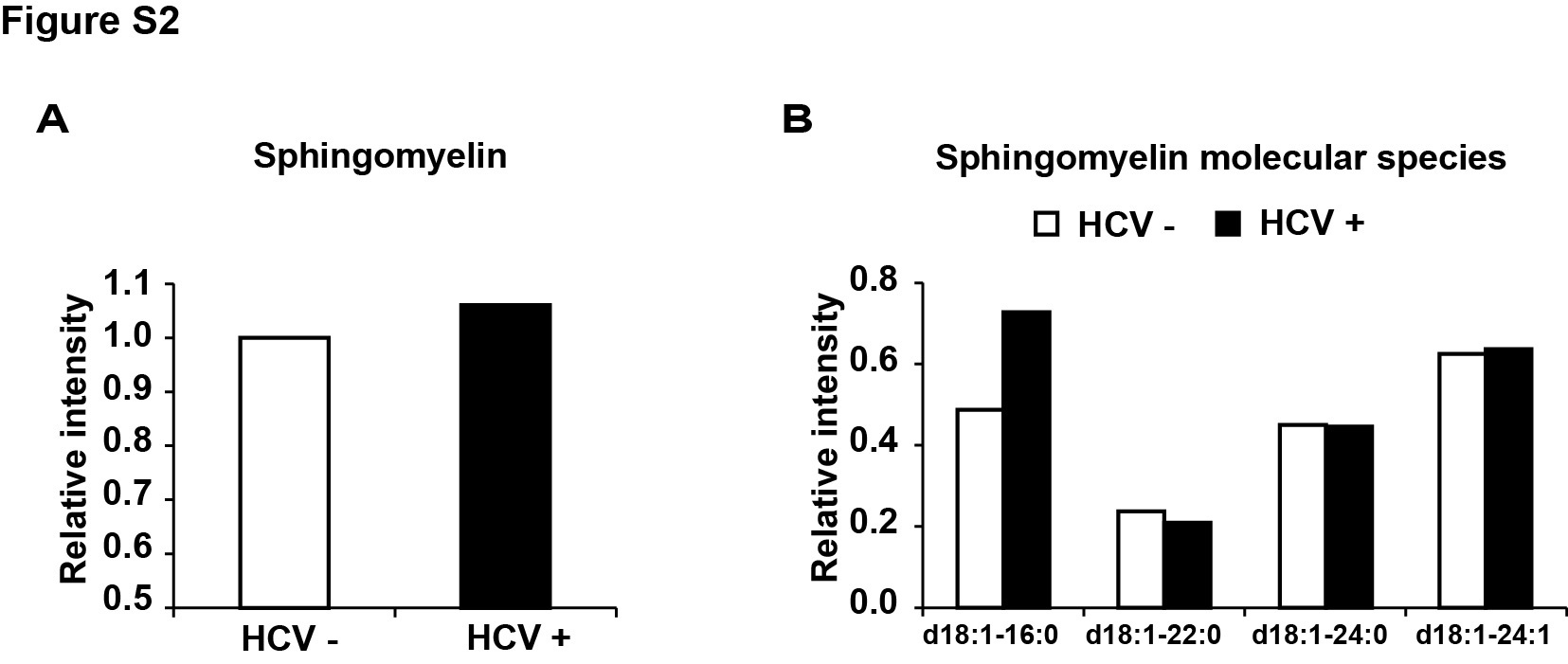

Supplement: Figure S2 — Effect of HCV infection in cultured cells. Comparison of the relative amounts of SM, as measured by MS analysis, in mock-infected (HuH-7 K4 cells) (white) and HCV (JFH-1)-infected cells (JFH/K4 cells) (black) (n = 1 per group). (JPG) [file ppat.1002860.s002.jpg]

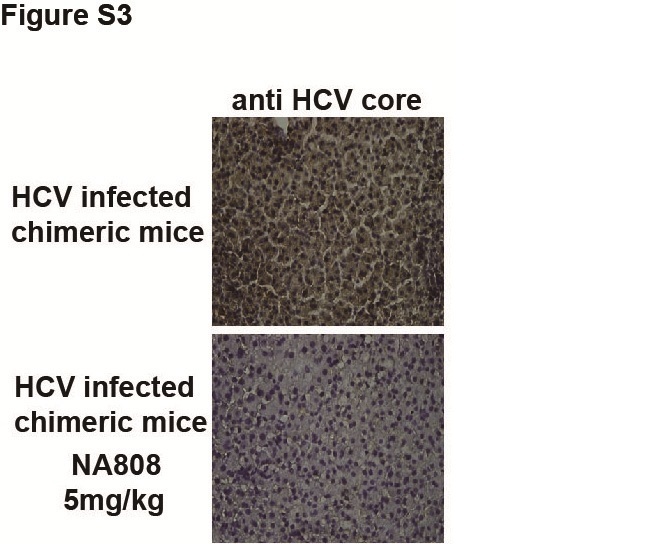

Supplement: Figure S3 — The expression of HCV core protein in HCV-infected chimeric mice. Histological analysis using immunohistochemical labeling of HCV core protein. (JPG) [file ppat.1002860.s003.jpg]

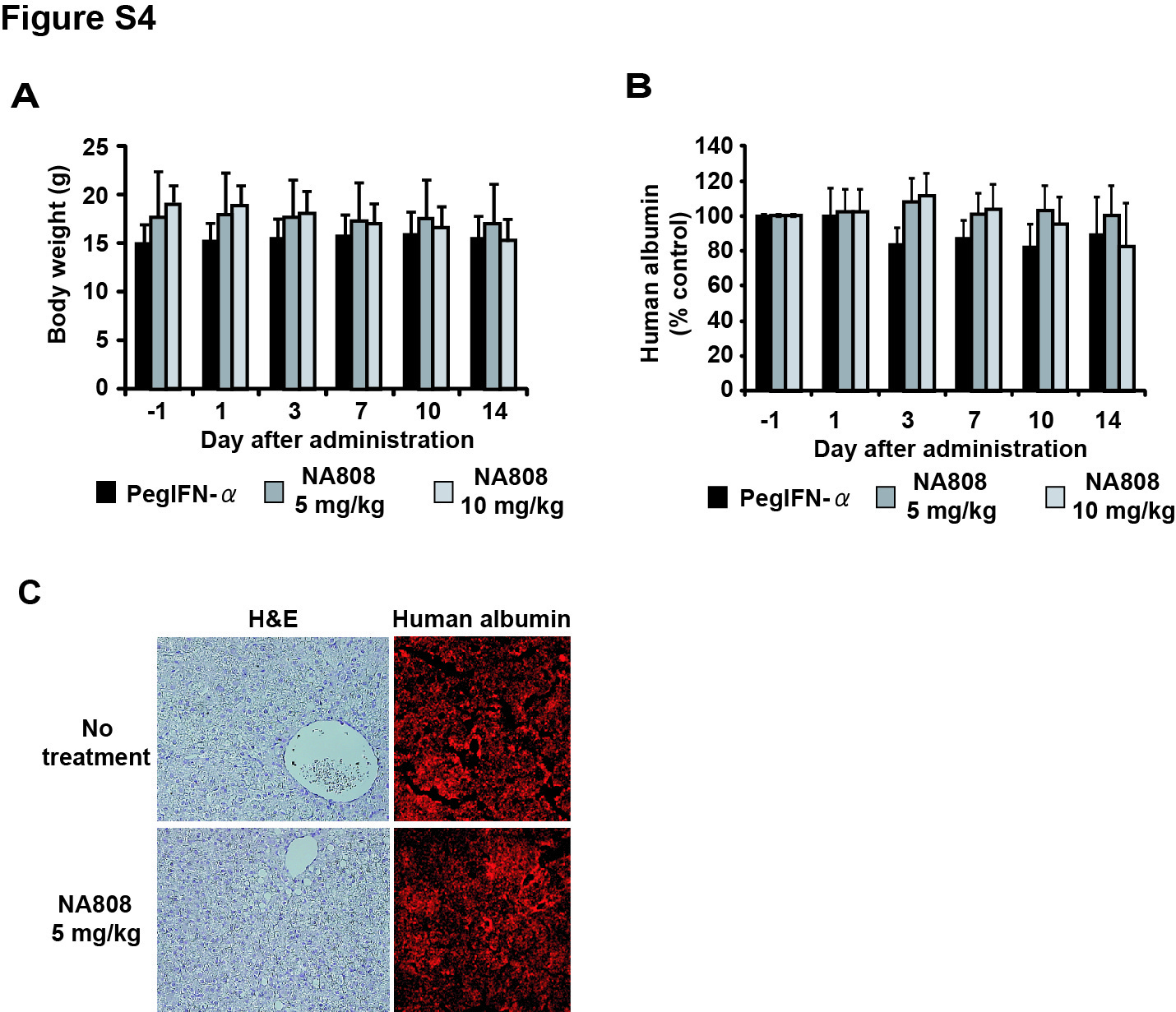

Supplement: Figure S4 — Effects of NA808 on HCV-infected chimeric mice. (A) Average body weight of mice during treatment. (B) Average human albumin concentrations in the sera of mice during treatment. (C) Histological analysis using H&E staining and immunofluorescent labeling of human albumin (red). In all cases, error bars indicate SDs. (JPG) [file ppat.1002860.s004.jpg]

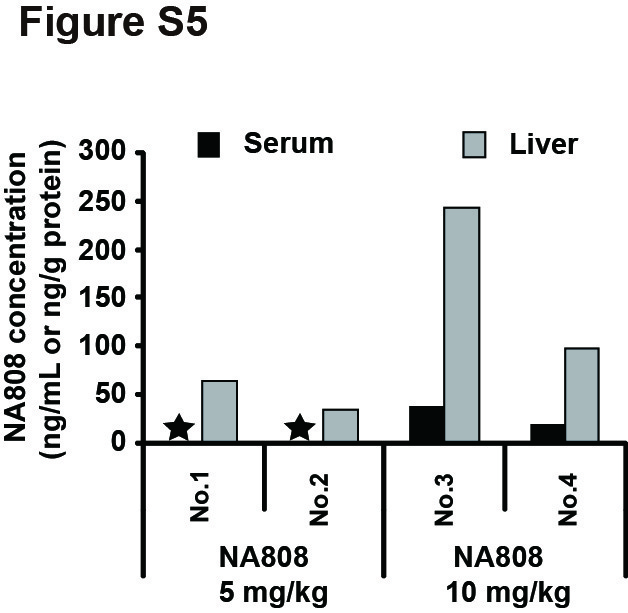

Supplement: Figure S5 — Concentrations of NA808 in chimeric mice receiving NA808 treatment. Concentration of NA808 in the liver (gray) and serum (black) of chimeric mice treated with 5 mg/kg or 10 mg/kg NA808. Stars indicate that NA808 level was not detected. (JPG) [file ppat.1002860.s005.jpg]

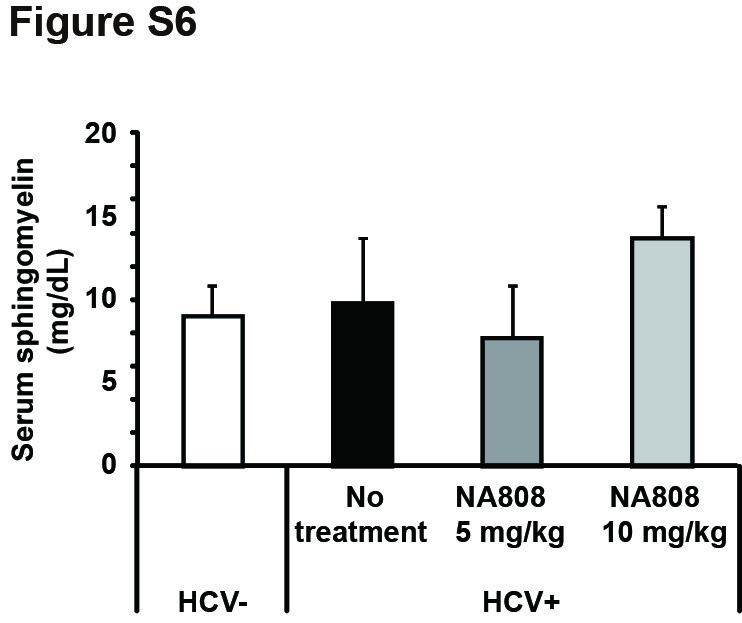

Supplement: Figure S6 — Sphingomyelin (SM) levels in the serum of chimeric mice receiving NA808 treatment. SM levels in the serum of chimeric mice (n = 3 per group) that were uninfected (HCV−), or infected (HCV+) but untreated or treated with 5 or 10 mg/kg NA808. Error bars indicate SDs. (JPG) [file ppat.1002860.s006.jpg]

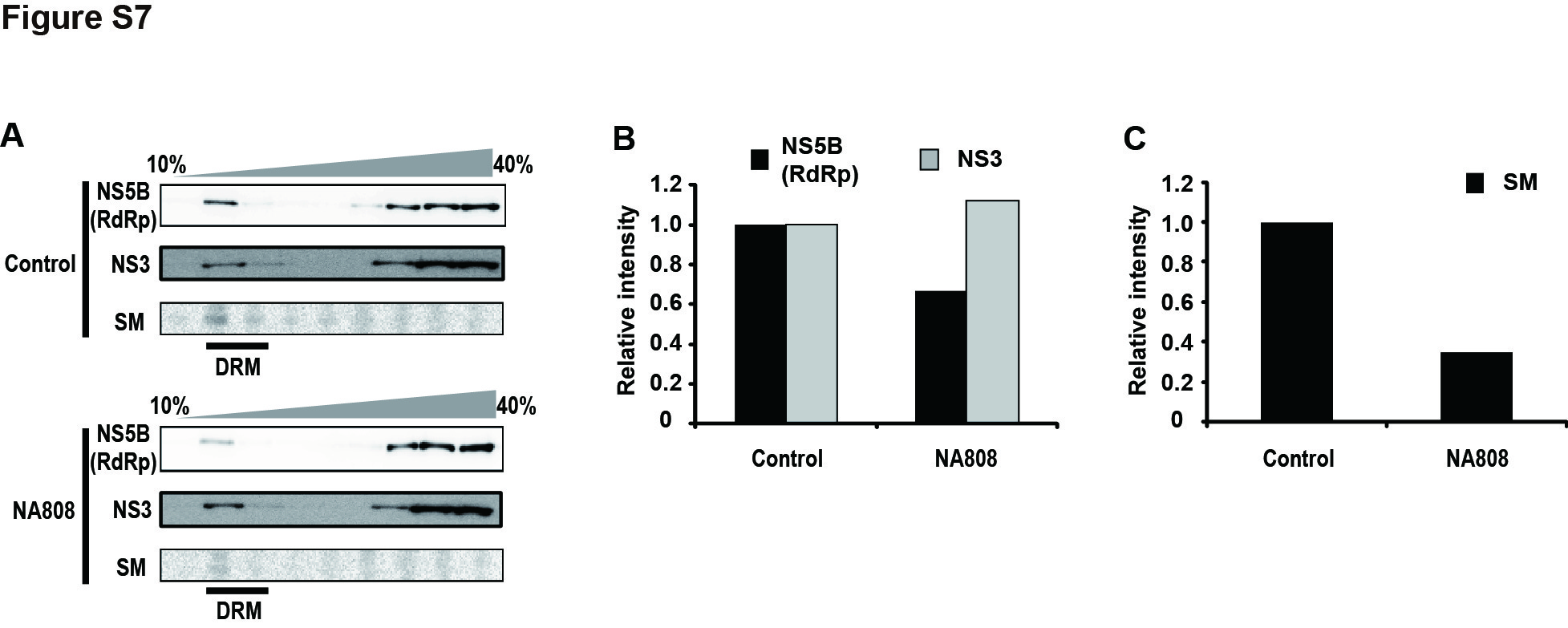

Supplement: Figure S7 — Effects of NA808 on associations between the HCV nonstructural 5B polymerase (RdRp) and sphingomyelin (SM). (A) Comparison of SDS-PAGE and TLC results for replicon cells receiving no treatment (Control) or NA808 treatment (NA808). NA808 dosage was 2.5 nM (for TLC) or 25 nM (for SDS-PAGE). (B) Relative band intensities of RdRp and NS3 in detergent-resistant membrane (DRM) fractions from cells receiving no treatment (Control) or 25 nM NA808 treatment (NA808). (C) Relative band intensities of SM in DRM fractions from cells receiving no treatment (Control) or 2.5 nM NA808 treatment (NA808). (JPG) [file ppat.1002860.s007.jpg]

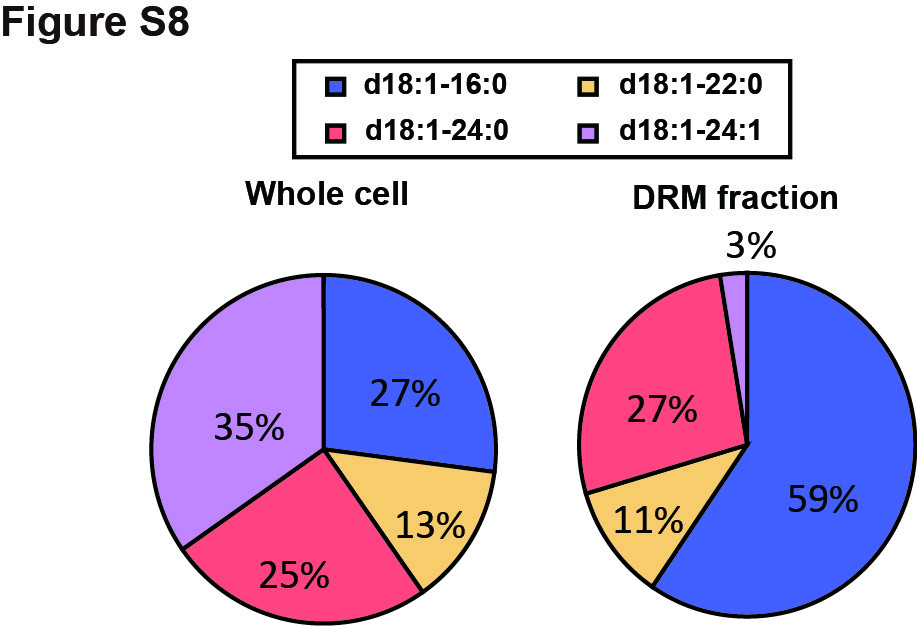

Supplement: Figure S8 — Composition ratio of SM molecular species in whole cells and DRM fraction of uninfected cells. (JPG) [file ppat.1002860.s008.jpg]

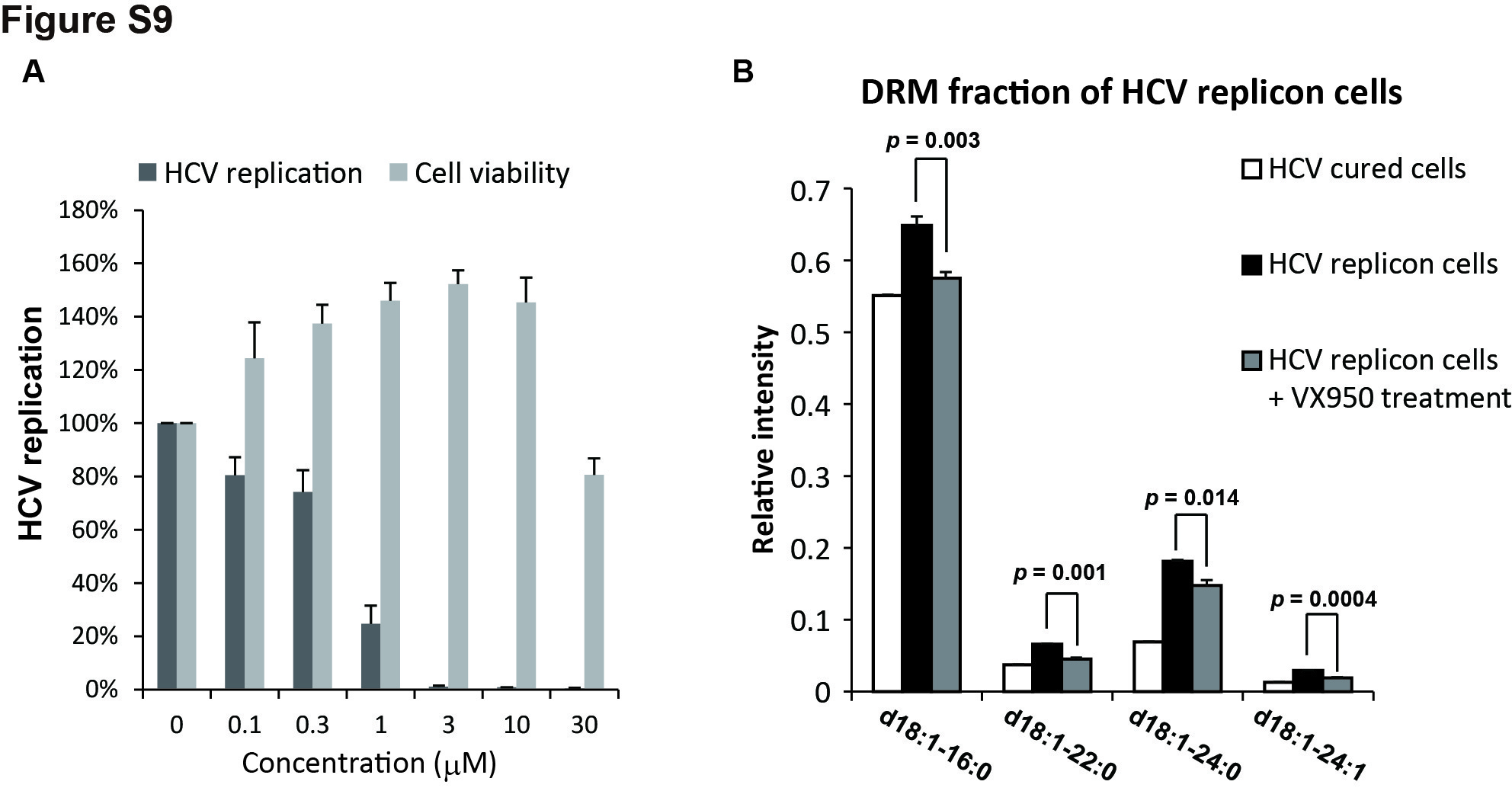

Supplement: Figure S9 — Effect of NS3 protease inhibitor on SM molecular species in the DRM fractions of subgenomic replicon cells. (A) Effect of NS3 protease inhibitor (VX950) on HCV replication (dark grey bars) and cell viability (light grey bars) in FLR3-1 replicon-containing cells. Error bars indicate SD. (B) Effect of NS3 protease inhibitor (VX950; 3 µM) on SM molecular species of DRM fractions of FLR 3-1 replicon-containing cells. Error bars indicate SDs. (JPG) [file ppat.1002860.s009.jpg]

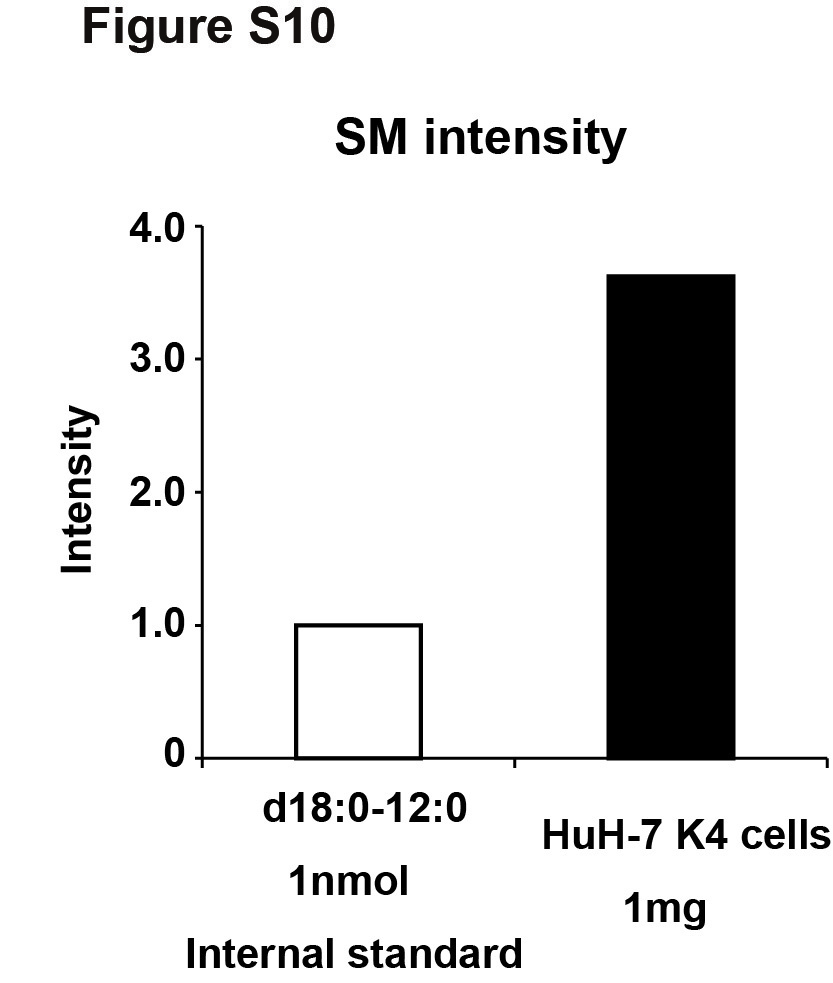

Supplement: Figure S10 — The estimated SM content in human hepatocytes. Left bar (white) indicates the intensity of SM internal standard (SM d18∶0-12∶0; 1 nmol) by mass spectrometer. Right bar indicates the intensity of 1 mg protein of human hepatocyte (HuH-7 K4). (JPG) [file ppat.1002860.s010.jpg]
