# Supplementary material for: Self-Enhancement of Hepatitis C Virus Replication by Promotion of Specific Sphingolipid Biosynthesis
Source: PLoS Pathog. 2012 Aug 16;8(8):e1002860. doi: 10.1371/journal.ppat.1002860 (PMC3420934; doi:10.1371/journal.ppat.1002860)
Supplement: Table S1 — Distribution of radioactivity in tissues after a single intravenous administration of [14C] NA808 at 2 mg/kg to non-fasting male rats. (PDF) [file ppat.1002860.s011.pdf]

Table S1

Tissue	Distribution of radioactivity (% of dose)			
	0.5 h	2 h	8 h	24 h
Cerebrum	0.00 ± 0.00	0.00 ± 0.00	0.00 ± 0.00	N.D.
Cerebellum	0.00 ± 0.00	0.00 ± 0.00	0.00 ± 0.00	0.00 ± 0.00
Pituitary gland	0.00 ± 0.00	N.D.	N.D.	N.D.
Eyeballs	0.00 ± 0.00	0.00 ± 0.00	0.00 ± 0.00	0.00 ± 0.00
Submaxillary gland	0.00 ± 0.00	0.00 ± 0.00	0.00 ± 0.00	0.00 ± 0.00
Thyroid	0.00 ± 0.00	N.D.	N.D.	N.D.
Thymus	0.00 ± 0.00	0.00 ± 0.00	0.00 ± 0.00	0.00 ± 0.00
Heart	0.00 ± 0.00	0.00 ± 0.00	0.00 ± 0.00	N.D.
Lung	0.02 ± 0.01	0.01 ± 0.00	0.00 ± 0.00	0.00 ± 0.00
Liver	16.77 ± 1.99	1.03 ± 0.69	0.10 ± 0.03	0.03 ± 0.01
Adrenal	0.00 ± 0.00	0.00 ± 0.00	0.00 ± 0.00	0.00 ± 0.00
Kidney	0.35 ± 0.03	0.21 ± 0.01	0.07 ± 0.02	0.03 ± 0.01
Spleen	0.00 ± 0.00	0.00 ± 0.00	0.00 ± 0.00	0.00 ± 0.00
Pancreas	0.01 ± 0.01	0.00 ± 0.00	0.00 ± 0.00	0.00 ± 0.00
Prostate	0.00 ± 0.00	0.00 ± 0.00	0.00 ± 0.00	0.00 ± 0.00
Testis	0.02 ± 0.00	0.01 ± 0.00	0.00 ± 0.00	0.00 ± 0.00
Epididymis	0.01 ± 0.01	0.00 ± 0.00	0.00 ± 0.00	0.00 ± 0.00
Seminal vesicle	0.00 ± 0.01	0.00 ± 0.00	0.00 ± 0.00	N.D.
Bladder	0.00 ± 0.00	0.00 ± 0.00	0.00 ± 0.00	0.00 ± 0.00
Stomach	0.15 ± 0.20	0.00 ± 0.00	0.00 ± 0.00	0.00 ± 0.00
Small intestine	4.44 ± 2.02	1.24 ± 0.68	0.06 ± 0.03	0.00 ± 0.01
Cecum	0.01 ± 0.00	0.00 ± 0.00	0.07 ± 0.01	0.00 ± 0.01
Large intestine	0.01 ± 0.00	0.00 ± 0.01	0.14 ± 0.07	0.00 ± 0.01

Each value represents the mean ± S.D.of three animals
N.D.: Not detected
