# Supplementary material for: Self-Enhancement of Hepatitis C Virus Replication by Promotion of Specific Sphingolipid Biosynthesis
Source: PLoS Pathog. 2012 Aug 16;8(8):e1002860. doi: 10.1371/journal.ppat.1002860 (PMC3420934; doi:10.1371/journal.ppat.1002860)
Supplement: Text S1 — Materials and methods for supporting information. Methods for “Infection of chimeric mice with hepatitis B virus”, “Quantification of human albumin”, “Histological staining and indirect immunofluorescence analysis”, and “Quantification of sphingomyelin (SM) in serum” are described. (DOCX) [file ppat.1002860.s013.docx]

**Text S1. Supporting Materials and Methods**

**Infection of chimeric mice with hepatitis B virus**

To demonstrate the hepatitis C virus (HCV) -specific nature of *SGMS-1/2* induction, infection of the transgenic mice (SCID/uPA) was repeated, but this time using hepatitis B virus (HBV). The model, including transplantation with human hepatocytes, was generated as described in the Materials and Methods section of the main text. The experiments covered in the supplemental figures used SCID/uPA animals, severe combined immunodeficiency (SCID) mice that are transgenic for additional copies of the urokinase plasminogen activator gene. This genotype results in apoptosis of mouse hepatocytes, allowing replacement with human hepatocytes (PhenixBio, Hiroshima, Japan). Six weeks after the transplantation of human hepatocytes, we intravenously injected each chimeric mouse with condensed culture supernatant containing HBV genotype C. By the time of the first drug administration, the serum HBV genomic DNA levels had reached 2.4 × 10^7^ to 1.2 × 10^9^ copies/mL. The protocols for the animal experiments were approved by the ethics committee of The Tokyo Metropolitan Institute of Medical Science.

**Quantification of human albumin**

Human albumin concentrations were measured in 2 μL of serum using the Alb-II kit (Eiken Chemical), according to the manufacturer’s instructions.

**Histological staining and indirect immunofluorescence analysis**

For histological analysis, liver sections were stained with hematoxylin and eosin (H&E). For immunohistochemistry, liver sections were probed by biotinylated anti-HCV core protein monoclonal antibody (BT515) as the primary antibody, followed by streptavidin-HRP, and DAB. Immunofluorescence analysis of liver sections was performed using anti-human albumin (Dako, Glostrup, Denmark) (primary antibody) followed by anti-goat IgG-Alexa-546 (secondary antibody).

**Quantification of sphingomyelin (SM) in serum**

We quantified serum SM levels with an SM assay kit (Cayman Chemical, Ann Arbor, MI, USA).
